# Comparative Effectiveness of 2 Next-Generation Scatter Radiation Shielding Systems

**DOI:** 10.1016/j.jscai.2025.103786

**Published:** 2025-07-07

**Authors:** Robert F. Riley, Stephen Kidd, Juliette Power, Brian Stegman, Thom G. Dahle

**Affiliations:** a Division of Cardiology, Overlake Medical Center, Bellevue, Washington; b Division of Cardiology, St. Cloud Hospital, St. Cloud, Minnesota

**Keywords:** catheterization laboratory, orthopedic injury, radiation protection

## Abstract

**Background:**

Scatter radiation is a health risk for personnel in x-ray–guided procedure rooms. This study compared the effectiveness of scatter radiation reduction from 2 next-generation radiation shielding systems.

**Methods:**

Vertical poles with mounted radiation survey meters were positioned at 6 points around a catheterization laboratory imaging table where procedural staff typically stand. Meters were mounted on vertical tracks where the sensor could be raised on the track with stops every 20 cm (up to 200 cm). Fluoroscopy (15 frames per second) was then performed on an anthropomorphic phantom with a cardiac silhouette in PA and 4 quadrant angulations using a Philips Allura C-arm with a 9-inch detector and ClarityIQ dose reduction software (both Philips). Scatter radiation measurements were reported in μSv/h under 3 radiation shielding conditions: no shielding, using the Rampart IC shielding system, and using the EggNest Complete shielding system (Egg Medical Inc).

**Results:**

Scatter radiation was not evenly distributed around the table, with higher doses noted at the head of the bed compared to the feet and on the left side of the table compared to the right. Positions correlating with the primary operator and assistant had similarly significant average reductions in scatter radiation for both novel protection systems (EggNest Complete 3 ± 7 μSv/h vs Rampart IC 10 ± 40 μSv/h; *P* = .18). However, the EggNest Complete system showed substantial reductions in scatter radiation measurements compared to the Rampart IC for positions at the head of the bed and the nursing position (26 ± 56 vs 131 ± 157 μSv/h; *P* < .01). These results were similar in all standard x-ray angulations.

**Conclusions:**

Compared to no shielding, both the EggNest Complete and the Rampart IC systems significantly lowered radiation measurements for the operator and assistant positions. However, the EggNest Complete system provided additional significant protection for the head of the bed and the nurse positions, which was not seen with the Rampart IC system.

## Introduction

Interventional cardiology procedures often involve prolonged exposure to ionizing radiation through the use of continuous fluoroscopy, which poses significant risks not only to patients but also to the health care professionals involved in performing these procedures.^1^, ^2^, ^3^, ^4^, ^5^ The increasing complexity of interventional techniques has heightened concerns regarding radiation dose management.^6^

Although lead shields and wearable aprons are often utilized as radiation barriers during these procedures, they function more like radiation filters, blocking 82% to 98% of x-ray photons, depending on the “lead equivalency” of the shield/apron.^7^ In addition to their imperfect radiation protection, wearable lead aprons can also result in significant orthopedic injuries, with the majority of interventional cardiologists reporting at least 1 major orthopedic injury during their career.^8^^,^^9^

There are several novel radiation protection systems that can reduce the exposure to scatter radiation for workers in these rooms.^10^, ^11^, ^12^ The EggNest Complete system (Egg Medical Inc) utilizes a series of shields and covers around and under the table to achieve a reduction in scatter radiation without adding additional potential for orthopedic injury.^13^ The Rampart IC system (Rampart IC) is a 1 mm lead equivalence shield on wheels with additional table shields.^12^ The purpose of this study was to compare scatter radiation levels at positions around a catheterization laboratory (cath lab) table where staff usually stand during procedures for each system compared to no shielding.

## Materials and methods

### Radiation measurement

Scatter radiation dose levels were measured using 6 solid-state survey meters (X2 Survey Sensor, RaySafe) that were all calibrated by the manufacturer within 6 months of the study. The range of detection for these meters is 0 μSv/h to 150 mSv/h, with a 95% CI of scatter radiation measurements previously shown to be ±3.3%.^14^ Therefore, a single measurement per experimental condition was considered sufficient. Scatter radiation dose levels were recorded as a dose rate (μSv/h).

Each survey meter was affixed to a holder mounted on a track in a calibrated pole, 2 m in height, where the survey meter could be raised on the track with stops at every 20 cm (20-200 cm). This setup ensured that the angle and vertical height of the survey meters were easily reproducible between measurement conditions. The poles were placed where personnel typically stand during procedures (Central Illustration). Position 3 next to the right chest was eliminated prior to starting the study because the Rampart IC system occupied that space; hence, measurements were unable to be obtained at that position for that device.^15^

**Central Illustration:**
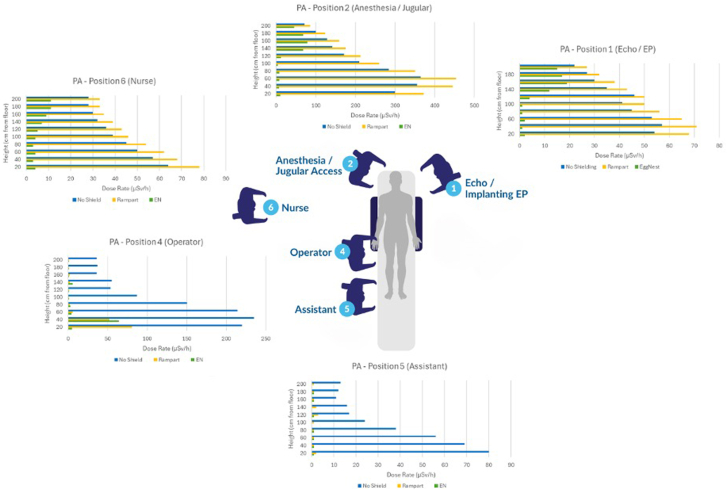
**Average radiation scatter measurements by positionaround the catheterization laboratory table at various heights in the PA angulation.**

### X-ray imaging

We used a whole-body anthropomorphic phantom obtained from the US Department of Energy Phantom Library (Model RESL 201). The anthropomorphic phantom generated scatter radiation that approximated a large human.^16^

Fluoroscopy (15 fps) for 10 seconds at each level was performed using a Philips Allura C-arm with a 9-inch detector and ClarityIQ x-ray dose reduction software (both Philips). The collimators were adjusted to the edge of the imaging field.

Table position was minimally adjusted to ensure that x-ray tube settings (kilovolts, milliamps, and pulse width) and air kerma were similar between each shielding condition. X-ray systems settings and output for each shielding condition are detailed in Supplemental Table S1.

### Shielding conditions

**Figure 1:**
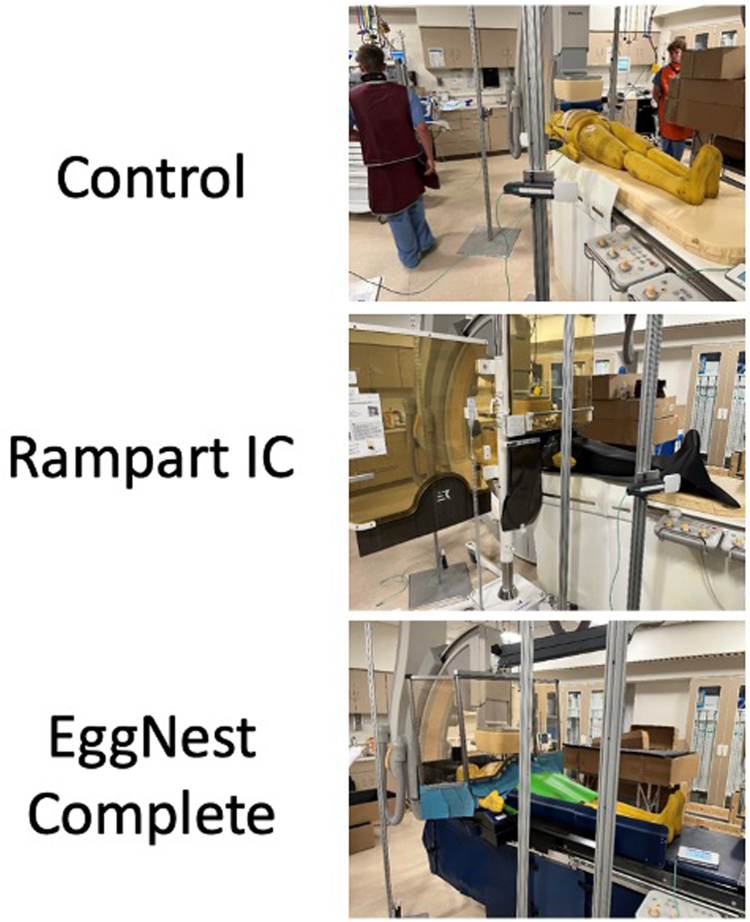
**The 3 shielding conditions evaluated in this study includes no shielding (control), Rampart IC shielding system, and the EggNest Complete shielding system**.

Scatter radiation measurements were measured under 3 shielding conditions: no shielding, using the Rampart IC system, and using the EggNest Complete radiation shielding system as seen in Figure 1.

The Rampart IC system consists of a 1 mm lead equivalence motorized shield on wheels which is primarily lead-impregnated acrylic and is contoured to the patient outline with flexible lead rubber flaps.^12^

The EggNest Complete shielding system consists of a carbon fiber–based platform, which is mounted onto the x-ray table. Flexible shielding (0.5 mm lead equivalent) below the table is affixed to the platform such that there is a radiation shield around the sides and head of the table that moves with the C-arm gantry. In addition, flip shields (0.5 mm lead equivalent) around the table can be rotated upwards after the patient is moved to the x-ray table to provide shielding around the patient. A ceiling or boom mounted clear acrylic shield with 1.0 mm lead equivalent shielding is placed over the patient, such that a cutout with a radiation shielding fringe is placed against the patient and extends across the arm. The right arm is held in a cradle with additional radiation shielding.

### Experimental protocol

The table with the phantom was positioned such that the phantom heart and the upper edge of the diaphragm were in the 9-inch imaging field. For each shielding condition, measurements at all 5 C-arm positions were taken in the following x-ray angulations: PA, RAO 30° caudal 20°, LAO 40° caudal 20°, RAO 30° cranial 20°, and LAO 30° cranial 20°.

### Statistical analysis

Average or summed values are expressed as mean ± SD. Differences in paired scatter radiation intensity measurements between each of the shielding conditions were analyzed with an analysis of variance. A *P* value < .05 was considered statistically significant.

## Results

### Effects of personnel location on scatter radiation and protection

**Figure 2:**
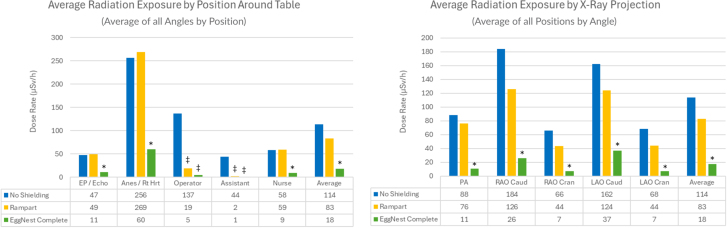
**Average radiation levels stratified by position and x-ray projection around the table.** ∗*P* < .05 vs Rampart IC and no shielding, ^‡^*P* < .05 vs no shielding.

Average scatter radiation measurements for each position around the x-ray lab table for each shielding condition in the PA angulation at various heights are shown in the Central Illustration. Average radiation levels for all x-ray angulations by position around the table can be seen in Figure 2, with average values for each angulation and each position in Table 1. The average scatter radiation level across angulations was highest without shielding (114 ± 49 μSv/h) and lowest using the EggNest Complete system (18 ± 13 μSv/h, *P* < .01 for EggNest Complete vs no shielding and Rampart IC). For all positions evaluated, position 2 (where personnel often stand for anesthesia or jugular access) had the highest overall exposure when evaluated without shielding, regardless of the type of shielding used. The average levels of scatter radiation at position 1 (echocardiographer/electrophysiologist position), position 2, and position 6 (where the nursing personnel often stand) were similar for no shielding and with Rampart IC shielding. Using the EggNest Complete system, however, there were substantial reductions in scatter radiation measurements compared to no shielding and the Rampart IC system for these positions (average of all 3 positions in all angulations: 27 ± 56 using the EggNest Complete vs 131 ± 157 μSv/h using the Rampart IC system, an 80% overall reduction when testing the EggNest Complete compared to Rampart IC, *P* < .01).

**Table 1:** Scatter radiation levels for all positions and x-ray angulations, by shielding type

	EP/Echo	Anesthesia/jugular access	Operator	Assistant	Nurse	Average
No shielding						
PA	41	213	112	34	41	88
RAO 30°/cranial 20°	33	147	85	30	37	66
LAO 30°/cranial 30°	29	147	97	31	38	68
RAO 30°/caudal 20°	–a	394	192	67	85	184
LAO 40°/caudal 20°	86	377	201	58	89	162
Mean ± SD	47 ± 23	256 ± 109	137 ± 49	44 ± 15	58 ± 24	114 ± 50
Rampart ic shielding						
PA	50	264	15	1	49	76
RAO 30°/cranial 20°	30	149	2	1	37	44
LAO 30°/cranial 20°	30	147	4	1	37	44
RAO 30°/caudal 20°	–a	399	19	3	84	126
LAO 40°/caudal 20°	88	387	56	3	87	124
Mean ± SD	49 ± 23	269 ± 110	19 ± 20^b^	2 ± 1^b^	59 ± 22	83 ± 37^b^
EggNest Complete shielding						
PA	7	31	9	1	6	11
RAO 30°/cranial 20°	8	21	1	1	5	7
LAO 30°/cranial 30°	3	25	2	1	5	7
RAO 30°/caudal 20°	–	82	5	2	15	26
LAO 40°/caudal 20°	26	139	6	1	12	37
Mean ± SD	11 ± 9^c^	60 ± 46^c^	5 ± 3^b^	1 ± 1^c^	9 ± 4^c^	18 ± 12^c^

Values are the average measurements from all heights at each position in µSv/h.

a The x-ray gantry blocked the measurement at position 1 in the RAO caudal angulation.

b *P* < .01 vs standard shielding.

c *P* < .01 vs Rampart IC and standard shielding.

At positions 4 (primary operator position) and 5 (assistant position), both the Rampart IC and the EggNest Complete systems markedly reduced radiation exposure to levels compared to no shielding (*P* < .01 vs no shielding for both). There was a modest difference in average measurements for these positions between EggNest Complete and the Rampart IC (average radiation dose for all angulations using EggNest Complete at the operator position was 5 ± 3 μSv/h vs 19 ± 20 μSv/h using Rampart IC, *P* = .06, and for the assistant position 1 ± 1 μSv/h vs 2 ± 1 μSv/h, respectively, *P* = .22).

### Effects of x-ray angulation on scatter radiation and protection

Scatter radiation measurements at each position around the table in each of the C-arm angulations, positions, and heights can be seen in Table 1, Figure 2, and Supplemental Table S2. These data show that in each of these standard x-ray C-arm angulations, the distribution of scatter radiation was largely similar to that seen in the PA position: radiation measurements were higher at the head of the bed compared to the foot of the bed (*P* < .01) and below the table compared to above the table (*P* < .01). Similarly, both Rampart IC and EggNest Complete provided similar protection for the operator and assistant positions (*P* = .06 for the primary operator and *P* = .18 for the assistant positions) in all C-arm angulations. However, the EggNest Complete shielding system provided significant reductions in radiation measurements for the positions at the head of the bed (positions 1 and 2) and the nurse position at all x-ray positions and heights compared to no shielding and Rampart IC (*P* < .01 for EggNest Complete compared to both Rampart IC and no shielding at all 3 positions).

## Discussion

This study compared the effectiveness of 2 novel, commercially available radiation protection systems, Rampart IC and EggNest Complete, on reducing scatter radiation in the cath lab when compared to no shielding. Measurements of scatter radiation distribution at baseline showed that scatter radiation was highest at the head of the table and below the table. When compared to no shielding, both the Rampart IC and the EggNest Complete systems provided significant protection for the primary operator and assistant positions on the right side of the table. However, the EggNest Complete system also provided significant radiation reduction for the positions at the head of the table (both sides) and for the nurse position, which was not seen with the Rampart IC system. These results were similar and consistent across all standard C-arm angulations and heights. These data show that the EggNest Complete system provided significant radiation protection for all positions around the table, whereas the Rampart IC system only provided protection for the positions caudal to the Rampart shield.

A fundamental principle of radiation protection is that personnel radiation doses should be as low as reasonably achievable. To achieve as low as reasonably achievable radiation doses and gain broad acceptance, these next-generation shielding systems must first demonstrate a significant reduction in radiation exposure compared to currently accepted standard shielding, which has been shown for both the EggNest Complete and Rampart IC systems for the primary operator and assistant positions.^15^^,^^17^ In addition, however, an ideal shielding system should provide protection to all members of the medical team, regardless of their position in the room. Next-generation shielding systems should also be to be scalable to provide protection for the various types of procedures performed in cath labs including endovascular procedures, ablations, device implants, interventional neurology procedures, interventional radiology procedures, and others. This type of versatility requires protection for all team members in the cath lab, regardless of their position in the room. This study illustrates that the EggNest Complete system can provide protection for the entire cath lab team, facilitating its use in a wide variety of procedures.

Angulation of the x-ray gantry can significantly change scatter radiation doses around the table. In this study, caudal angulations significantly increased total scatter radiation in the room compared to noncaudal views. The unequal distribution of scatter radiation seen in this study reemphasizes the importance of having a radiation protection system that protects all positions around the bed at all heights and x-ray gantry positions in order to provide consistent protection for all members of the cath lab team involved in the diverse types of procedures performed in today’s modern cath lab.

There were several limitations to this study. First, there is no currently accepted protocol for measuring scatter radiation protection in the cath lab. Although this study presents a rigorous design, there have been a variety of other study designs utilized for measuring scatter radiation without a currently accepted standard method.^10^, ^11^, ^12^ Additionally, we recorded radiation scatter in μSv/h as this is the output measure for the radiation sensor we utilized for the study (RaySafe). However, other studies have utilized other scatter measurement units (mRem). This again speaks to the need for a formalized standardized method for conducting these studies. Second, these measurements were recorded using an anthropomorphic human-shaped phantom. Although these measurements only approximate the scatter from a large human, the potential advantage of using a phantom is that very detailed radiation measurements can be reproducibly obtained with excellent spatial resolution. Third, x-ray output varies between x-ray systems. In this study, we used a low-dose system with image optimization software (ClarityIQ). The absolute scatter radiation dose may be different with other systems but the distribution and relative scatter radiation levels should be similar. Finally, there is variability in other types of shielding for positions at the head of the bed in some procedures (barrier shielding for some imagers and anesthesiologists, etc), which could further attenuate radiation protection at these positions and thus, the results of this study. The results from this study will need to be confirmed in further clinical studies.

## Conclusion

Compared to no shielding, both the EggNest Complete and the Rampart IC systems significantly lowered radiation measurements for the operator and assistant positions. However, the EggNest Complete system provided additional significant radiation protection for cath lab team members at the head of the bed and nursing positions, which was not seen with the Rampart IC system. Utilizing a radiation protection system that provides protection for all members of the cath lab team is key to ensuring the scalability of a protection system that can be utilized for the multitude of procedures performed in today’s modern cath lab.
