# Supplementary material for: Comparative Effectiveness of 2 Next-Generation Scatter Radiation Shielding Systems
Source: J Soc Cardiovasc Angiogr Interv. 2025 Jul 7;4(8):103786. doi: 10.1016/j.jscai.2025.103786 (PMC12462136; doi:10.1016/j.jscai.2025.103786)
Supplement: Supplementary Table 1 [file mmc1.docx]

**Supplemental Table 1:** X-ray imaging parameters for each shielding condition

|  | **No Shielding** | **Rampart IC** | **EggNest Complete** |  |
| --- | --- | --- | --- | --- |
| **PA Projection** |  |  |  |  |
| mA | 6.8 | 6.8 | 6.7 |  |
| kV | 75 | 74 | 74 |  |
| Pulse width (ms) | 4 | 4 | 4 |  |
| **RAO 30/Cran 20** |  |  |  |  |
| mA | 7.4 | 7.4 | 7.4 |  |
| kV | 72 | 72 | 72 |  |
| Pulse width (ms) | 4 | 4 | 4 |  |
| **LAO 30/Cran 30** |  |  |  |  |
| mA | 7.3 | 7.5 | 7.5 |  |
| kV | 72 | 71 | 73 |  |
| Pulse width (ms) | 4 | 4 | 4 |  |
| **RAO 30/Caud 20** |  |  |  |  |
| mA | 9.4 | 9.1 | 9.1 |  |
| kV | 80 | 80 | 80 |  |
| Pulse width (ms) | 5 | 5 | 5 |  |
| **LAO 40/Caud 20** |  |  |  |  |
| mA | 9.2 | 9.1 | 9.1 |  |
| kV | 78 | 78 | 79 |  |
| Pulse width (ms) | 5 | 5 | 5 |  |
| **Average±SD of all angles** |  |  |  | **p value** |
| **mA** | **8.0±1.1** | **8.0±0.9** | **8.0±1.0** | **0.68** |
| **kV** | **75.4±3.2** | **75.0±3.5** | **75.6±3.3** | **0.27** |
| **Pulse width (ms)** | **4.4±0.5** | **4.4±0.5** | **4.4±0.5** | ***** |

* values were identical for each condition
