# Supplementary material for: Comparative Effectiveness of 2 Next-Generation Scatter Radiation Shielding Systems
Source: J Soc Cardiovasc Angiogr Interv. 2025 Jul 7;4(8):103786. doi: 10.1016/j.jscai.2025.103786 (PMC12462136; doi:10.1016/j.jscai.2025.103786)
Supplement: Supplementary Table 2 [file mmc2.docx]

RAO 30 CRANIAL 20

| **Height (cm from floor)** | **20** | **40** | **60** | **80** | **100** | **120** | **140** | **160** | **180** | **200** | **AVG** |  | **Average Radiation Dose Rate (µSv/h)** | |
| --- | --- | --- | --- | --- | --- | --- | --- | --- | --- | --- | --- | --- | --- | --- |
| **RAO30/Cran20 Position 1 (Echo / EP Implanter)** | | | | | | | | | | | |  | **Anesthesia / Echo / EP** | |
| **EggNest** |  |  |  | **1.7** | **1.2** | **2** | **8** | **13** | **14** | **14** | **8** |  | **EggNest Complete** | **15*** |
| **Rampart** |  |  |  | **47** | **42** | **36** | **29** | **22** | **19** | **17** | **30** |  | **Rampart IC** | **90** |
| **No Shielding** |  |  |  | **48** | **48** | **38** | **34** | **27** | **21** | **18** | **33** |  | **No Shielding** | **90** |
| **RAO30/Cran20 Position 2 (Anesthesia / Jugular Access)** | | | | | | | | | | | |  | **Operator / Assistant** | |
| **EggNest** | **20** | **9** | **8** | **8** | **4** | **8** | **69** | **51** | **22** | **10** | **21** |  | **EggNest Complete** | **1^** |
| **Rampart** | **222** | **265** | **298** | **254** | **182** | **123** | **100** | **28** | **9** | **4** | **149** |  | **Rampart IC** | **2^** |
| **No Shielding** | **221** | **262** | **297** | **253** | **183** | **122** | **93** | **23** | **8** | **4** | **147** |  | **No Shielding** | **58** |
| **RAO30/Cran20 Position 4 (Operator)** | | | | | | | | | | | |  | **Nurse** | |
| **EggNest** | **3** | **1.5** | **2** | **1.9** | **0.8** | **1.1** | **0.8** | **1** | **0.8** | **0.7** | **1** |  | **EggNest Complete** | **5*** |
| **Rampart** | **4** | **3** | **2** | **1** | **1** | **2** | **1** | **1** | **1** | **1** | **2** |  | **Rampart IC** | **37** |
| **No Shielding** | **11** | **45** | **280** | **214** | **121** | **42** | **50** | **39** | **28** | **20** | **85** |  | **No Shielding** | **37** |
| **RAO30/Cran20 Position 5 (Assistant)** | | | | | | | | | | | |  | ***p<0.01 vs Rampart IC and No Shielding** | |
| **EggNest** | **1.1** | **0.3** | **0.3** | **1.7** | **1.4** | **0.8** | **0.7** | **0.5** | **0.8** | **0.5** | **1** |  | **^p<0.01 vs No Shielding** | |
| **Rampart** | **1** | **1** | **1** | **1** | **1** | **1** | **1** | **1** | **1** | **1** | **1** |  |  |  |
| **No Shielding** | **79** | **71** | **55** | **34** | **20** | **8** | **9** | **7** | **7** | **7** | **30** |  |  |  |
| **RAO30/Cran20 Position 6 (Nurse)** | | | | | | | | | | | |  |  |  |
| **EggNest** | **3** | **3** | **3** | **3** | **3** | **5** | **6** | **8** | **9** | **9** | **5** |  |  |  |
| **Rampart** | **57** | **54** | **49** | **42** | **36** | **32** | **29** | **25** | **24** | **22** | **37** |  |  |  |
| **No Shielding** | **57** | **56** | **50** | **42** | **37** | **33** | **29** | **26** | **23** | **21** | **37** |  |  |  |
| Dose Rates in µSv/h | | | | | | | | | | | |  |  |  |

RAO 30 CAUDAL 20

| **Height (cm from floor)** | **20** | **40** | **60** | **80** | **100** | **120** | **140** | **160** | **180** | **200** | **AVG** |  | **Average Radiation Dose Rate (µSv/h)** | |
| --- | --- | --- | --- | --- | --- | --- | --- | --- | --- | --- | --- | --- | --- | --- |
| **RAO30/Caud20 Position 1 (Echo / EP Implanter)** | | | | | | | | | | | |  | **Anesthesia / Echo / EP** | |
| **EggNest** | **The x-ray gantry blocked the measurement at Position 1  in the RAO Caudal angulation** | | | | | | | | | | |  | **EggNest Complete** | **82*** |
| **Rampart** |  |  |  |  |  |  |  |  |  |  |  |  | **Rampart IC** | **399** |
| **No Shielding** |  |  |  |  |  |  |  |  |  |  |  |  | **No Shielding** | **394** |
| **RAO30/Caud20 Position 2 (Anesthesia / Jugular Access)** | | | | | | | | | | | |  | **Operator / Assistant** | |
| **EggNest** | **34** | **22** | **20** | **21** | **21** | **31** | **275** | **199** | **188** | **10** | **82** |  | **EggNest Complete** | **4^** |
| **Rampart** | **529** | **650** | **682** | **561** | **478** | **387** | **286** | **240** | **172** | **6** | **399** |  | **Rampart IC** | **11^** |
| **No Shielding** | **518** | **632** | **678** | **560** | **473** | **390** | **280** | **235** | **168** | **6** | **394** |  | **No Shielding** | **129** |
| **RAO30/Caud20 Position 4 (Operator)** | | | | | | | | | | | |  | **Nurse** | |
| **EggNest** | **15** | **8** | **6** | **5** | **2** | **2** | **1.4** | **0.9** | **2** | **3** | **5** |  | **EggNest Complete** | **15*** |
| **Rampart** | **100** | **60** | **9** | **5** | **3** | **2** | **2** | **1.2** | **2** | **1.8** | **19** |  | **Rampart IC** | **84** |
| **No Shielding** | **360** | **410** | **414** | **308** | **178** | **94** | **77** | **39** | **23** | **15** | **192** |  | **No Shielding** | **85** |
| **RAO30/Caud20 Position 5 (Assistant)** | | | | | | | | | | | |  | ***p<0.01 vs Rampart IC and No Shielding** | |
| **EggNest** | **2** | **1.5** | **1.4** | **1.6** | **6** | **1.3** | **1.4** | **1.7** | **1.8** | **1.4** | **2** |  | **^p<0.01 vs No Shielding** | |
| **Rampart** | **4** | **3** | **2** | **2** | **2** | **4** | **3** | **3** | **2** | **3** | **3** |  |  |  |
| **No Shielding** | **151** | **148** | **127** | **84** | **52** | **29** | **28** | **19** | **17** | **14** | **67** |  |  |  |
| **RAO30/Caud20 Position 6 (Nurse)** | | | | | | | | | | | |  |  |  |
| **EggNest** | **7** | **8** | **9** | **7** | **10** | **13** | **18** | **23** | **30** | **29** | **15** |  |  |  |
| **Rampart** | **125** | **120** | **108** | **95** | **82** | **75** | **66** | **59** | **54** | **51** | **84** |  |  |  |
| **No Shielding** | **125** | **121** | **111** | **97** | **83** | **77** | **66** | **61** | **54** | **52** | **85** |  |  |  |
| Dose Rates in µSv/h | | | | | | | | | | | |  |  |  |

LAO 40 CAUDAL 20

| **Height (cm from floor)** | **20** | **40** | **60** | **80** | **100** | **120** | **140** | **160** | **180** | **200** | **AVG** |  | **Average Radiation Dose Rate (µSv/h)** | |
| --- | --- | --- | --- | --- | --- | --- | --- | --- | --- | --- | --- | --- | --- | --- |
| **LAO40/Caud20 Position 1 (Echo / EP Implanter)** | | | | | | | | | | | |  | **Anesthesia / Echo / EP** | |
| **EggNest** | **7** | **63** | **15** | **15** | **6** | **5** | **12** | **25** | **51** | **64** | **26** |  | **EggNest Complete** | **83*** |
| **Rampart** | **7** | **110** | **160** | **136** | **123** | **102** | **84** | **64** | **50** | **39** | **88** |  | **Rampart IC** | **237** |
| **No Shielding** | **7** | **93** | **161** | **138** | **125** | **102** | **83** | **64** | **48** | **38** | **86** |  | **No Shielding** | **231** |
| **LAO40/Caud20 Position 2 (Anesthesia / Jugular Access)** | | | | | | | | | | | |  | **Operator / Assistant** | |
| **EggNest** |  |  | **20** | **36** | **11** | **21** | **128** | **370** | **308** | **220** | **139** |  | **EggNest Complete** | **4^** |
| **Rampart** |  |  | **335** | **875** | **648** | **484** | **305** | **200** | **142** | **105** | **387** |  | **Rampart IC** | **30^** |
| **No Shielding** |  |  | **217** | **898** | **631** | **502** | **317** | **204** | **144** | **106** | **377** |  | **No Shielding** | **130** |
| **LAO40/Caud20 Position 4 (Operator)** | | | | | | | | | | | |  | **Nurse** | |
| **EggNest** | **16** | **11** | **11** | **11** | **3** | **3** | **2** | **2** | **1** | **1** | **6** |  | **EggNest Complete** | **12*** |
| **Rampart** | **273** | **263** | **12** | **4** | **1** | **1** | **2** | **2** | **1** | **2** | **56** |  | **Rampart IC** | **87** |
| **No Shielding** | **350** | **384** | **360** | **285** | **183** | **101** | **100** | **92** | **82** | **71** | **201** |  | **No Shielding** | **89** |
| **LAO40/Caud20 Position 5 (Assistant)** | | | | | | | | | | | |  | ***p<0.01 vs Rampart IC and No Shielding** | |
| **EggNest** | **2** | **2** | **2** | **1** | **1** | **1** | **2** | **1** | **1** | **1** | **1** |  | **^p<0.01 vs No Shielding** | |
| **Rampart** | **5** | **7** | **2** | **1** | **1** | **6** | **2** | **2** | **2** | **1** | **3** |  |  |  |
| **No Shielding** | **120** | **114** | **95** | **63** | **44** | **32** | **27** | **28** | **28** | **27** | **58** |  |  |  |
| **LAO40/Caud20 Position 6 (Nurse)** | | | | | | | | | | | |  |  |  |
| **EggNest** | **10** | **12** | **10** | **7** | **8** | **10** | **12** | **15** | **17** | **20** | **12** |  |  |  |
| **Rampart** | **140** | **132** | **120** | **104** | **84** | **80** | **70** | **56** | **45** | **40** | **87** |  |  |  |
| **No Shielding** | **140** | **136** | **122** | **107** | **86** | **82** | **72** | **57** | **47** | **41** | **89** |  |  |  |
| Dose Rates in µSv/h | | | | | | | | | | | |  |  |  |

LAO 30 CRANIAL 20

| **Height (cm from floor)** | **20** | **40** | **60** | **80** | **100** | **120** | **140** | **160** | **180** | **200** | **AVG** |  | **Average Radiation Dose Rate (µSv/h)** | |
| --- | --- | --- | --- | --- | --- | --- | --- | --- | --- | --- | --- | --- | --- | --- |
| **LAO30/Cran20 Position 1 (Echo / EP Implanter)** | | | | | | | | | | | |  | **Anesthesia / Echo / EP** | |
| **EggNest** | **2** | **2** | **2** | **2** | **2** | **3** | **6** | **4** |  |  | **3** |  | **EggNest Complete** | **14*** |
| **Rampart** | **39** | **44** | **45** | **36** | **29** | **25** | **15** | **5** |  |  | **30** |  | **Rampart IC** | **89** |
| **No Shielding** | **38** | **42** | **43** | **35** | **29** | **25** | **16** | **5** |  |  | **29** |  | **No Shielding** | **88** |
| **LAO30/Cran20 Position 2 (Anesthesia / Jugular Access)** | | | | | | | | | | | |  | **Operator / Assistant** | |
| **EggNest** | **6** | **4** | **6** | **9** | **2** | **8** | **49** | **61** | **57** | **45** | **25** |  | **EggNest Complete** | **1^** |
| **Rampart** | **134** | **269** | **295** | **231** | **157** | **112** | **84** | **74** | **67** | **51** | **147** |  | **Rampart IC** | **3^** |
| **No Shielding** | **127** | **272** | **298** | **233** | **161** | **114** | **85** | **59** | **69** | **53** | **147** |  | **No Shielding** | **64** |
| **LAO30/Cran20 Position 4 (Operator)** | | | | | | | | | | | |  | **Nurse** | |
| **EggNest** | **6** | **3** | **2** | **2** | **2** | **2** | **1** | **1** | **1** | **1** | **2** |  | **EggNest Complete** | **5*** |
| **Rampart** | **21** | **13** | **2** | **2** | **1** | **1** | **1** | **1** | **1** | **1** | **4** |  | **Rampart IC** | **37** |
| **No Shielding** | **194** | **215** | **206** | **140** | **78** | **39** | **31** | **22** | **21** | **20** | **97** |  | **No Shielding** | **38** |
| **LAO30/Cran20 Position 5 (Assistant)** | | | | | | | | | | | |  | ***p<0.01 vs Rampart IC and No Shielding** | |
| **EggNest** | **0** | **0** | **0** | **0** | **1** | **1** | **0** | **1** | **1** | **1** | **1** |  | **^p<0.01 vs No Shielding** | |
| **Rampart** | **1** | **0** | **1** | **0** | **0** | **3** | **2** | **1** | **1** | **0** | **1** |  |  |  |
| **No Shielding** | **23** | **90** | **75** | **42** | **26** | **12** | **12** | **13** | **10** | **10** | **31** |  |  |  |
| **LAO30/Cran20 Position 6 (Nurse)** | | | | | | | | | | | |  |  |  |
| **EggNest** | **3** | **4** | **5** | **3** | **3** | **4** | **6** | **7** | **7** | **8** | **5** |  |  |  |
| **Rampart** | **63** | **58** | **51** | **45** | **37** | **30** | **27** | **22** | **18** | **16** | **37** |  |  |  |
| **No Shielding** | **65** | **60** | **52** | **46** | **39** | **32** | **28** | **23** | **19** | **17** | **38** |  |  |  |
| **Dose Rates in µSv/h** | | | | | | | | | | | |  |  |  |
